# The Significance of Genetic Relatedness and Nest Sharing on the Worker‐Worker Similarity of Gut Bacterial Microbiome and Cuticular Hydrocarbon Profile in a Sweat Bee

**DOI:** 10.1002/ece3.71519

**Published:** 2025-06-09

**Authors:** Federico Ronchetti, Thomas Schmitt, Alexander Keller, Andrés García‐Reina, Pilar De la Rua, Ingolf Steffan‐Dewenter, Carlo Polidori

**Affiliations:** ^1^ Department of Animal Ecology and Tropical Biology, Biocentre University of Würzburg Würzburg Germany; ^2^ Cellular and Organismic Networks, Faculty of Biology Ludwig‐Maximilians‐Universität Munich Planegg Martinsried Germany; ^3^ Department of Zoology and Physical Anthropology University of Murcia Murcia Spain; ^4^ Department of Environmental Science and Policy (ESP) University of Milan Milan Italy

**Keywords:** bacteria, *Halictus scabiosae*, hydrocarbons, microsatellites, primitively eusocial bee

## Abstract

The cuticular hydrocarbon (CHC) profile and the gut microbiome (GM) are crucial traits which have a significant impact on the life of bees. In honey bees, the CHC profile and the GM interact finely through trophallaxis, such that the characteristics of the GM are partially defined by the chemical recognition among sisters. However, most of the known primitively eusocial bees show simpler social traits, including moderate genetic relatedness among colony members, often due to workers' nest drifting or dispersal, and lack of trophallaxis. Hence, primitively eusocial bees offer a great opportunity to evaluate the respective role of worker‐worker genetic relatedness and of the environment in which the adult lives (residency nest) on the interaction between CHC profile and GM. Here, we investigated such relationships in the primitively eusocial digger bee *Halictus scabiosae* (Halictidae). We found a high rate of nest‐drifting by workers, which leads to a consequent highly variable intra‐colonial genetic relatedness. Genetically closely related workers, even occupying distant nests, did possess both a more similar microbiome profile and a more similar CHC profile. Additionally, sharing the same nest seemed to account for the similarity of both CHC profile and GM among workers. Interestingly, differences in microbiome profile and in CHC profile were highly and positively correlated across workers, even after controlling for genetic relatedness. The results of our study point towards an impact of genetic relatedness on the GM and the CHC profile, but also suggest that microbiome and CHC profile are partially acquired through adult nest environment, and that microbiome possibly has a role in shaping the cuticular chemistry.

## Graphical abstract

The relationship between cuticular hydrocarbon (CHC) profile and the gut microbiome (GM) is poorly known in bees. In the primitively eusocial bee *Halictus scabiosae* we found a high rate of nest‐drifting by workers, which leads to a consequent highly variable intra‐colonial genetic relatedness. Genetically closely related workers, even occupying distant nests, did possess both a more similar microbiome profile and a more similar CHC profile, and sharing the same nest seemed to also account for the similarity of both CHC profile and GM among workers.

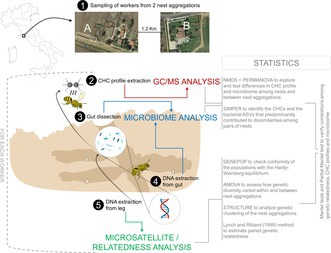

## 1 Introduction

Two of the traits heavily regulating the life of bees are the cuticular hydrocarbon (CHC) profile and the gut microbiome (GM). The CHCs cover the whole insect body as a thin layer which consists of a complex mixture of mainly *n*‐alkanes, alkenes, and methyl‐branched alkanes (Blomquist and Bagnères 2010). Because of its hydrophobic nature, this layer has the primary function of preventing desiccation, abrasion, and microbial infestation (Gibbs 1998). The CHCs have also evolved a variety of communicative functions, being largely used by insects in both intraspecific contexts, such as sexual interactions and nestmate recognition, and interspecific contexts such as recognition of natural enemies or recognition of prey or hosts (reviewed in Blomquist and Bagnères 2010; Kather and Martin 2015; Ayasse et al. 2001). In fact, one important property of a CHC profile is its species‐specificity. It is often also sex‐specific and, in social insects, colony specific (Blomquist and Bagnères 2010).

The GM is involved in a number of crucial functions, such as the development of the host (Chouaia et al. 2012), host nutrition (Bonilla‐Rosso and Engel 2018; Dharampal et al. 2020; Leonhardt et al. 2022), the regulation of reproduction (Hosokawa et al. 2010), and improving the immune system (Kaltenpoth 2009; Kwong et al. 2017). The GM in insects can be composed of protists, fungi, archaea, and bacteria. However, bacteria build the largest fraction of these organisms in the gut of insects (Engel and Moran 2013; Jing et al. 2020). The GM varies across species in total abundance of bacteria, their community composition, and their locations in the gut (e.g., Martinson et al. 2012; Ren et al. 2007).

The relevance of CHC profile and GM in insect ecology and evolution led to an increased amount of studies on both of these traits in the last two decades (e.g., Howard and Blomquist 2005; Blomquist and Bagnères 2010; Kather and Martin 2015; Leonhardt et al. 2016; Voulgari‐Kokota, McFrederick, et al. 2019; Voulgari‐Kokota, Grimmer, et al. 2019; Voulgari‐Kokota, Ankenbrand, et al. 2019; Sprenger and Menzel 2020; Engel and Moran 2013; Jing et al. 2020; Douglas 2018; Raymann and Moran 2018; Smith and Liebig 2017). However, in only very few cases have they been analyzed simultaneously (Mayr et al. 2021). This is a severe knowledge gap since the few available studies showed that chemical communication may also be influenced by the GM (Ezenwa and Williams 2014; Engl and Kaltenpoth 2018; Vernier et al. 2020; García‐Roa et al. 2022; Liberti et al. 2022). Firstly, there are studies reporting that the GM can cause changes in host metabolism that lead to a cascade of effects in the production of chemical signals (Ezenwa and Williams 2014; Engl and Kaltenpoth 2018). Secondly, a recent study on *Drosophila melanogaster* Meigen (Diptera) highlighted the influence of genetic relatedness on both the microbiota diversity and CHC profiles (García‐Roa et al. 2022). Thirdly, in highly eusocial species such as the Western honey bee ( *Apis mellifera* L.), it was shown that chemical recognition signals are in part defined by the shared microbial community within the colony's members (Vernier et al. 2020), with the GM altering the social network of honey bees (Liberti et al. 2022). Such a link between CHC profile and GM fits with the concept of a “holobiont”, i.e., individuals are composed of the host and its microbiome (Zilber‐Rosenberg and Rosenberg 2008) and hence the variations in CHCs may result from variations in microbiomes rather than only from individual genetics or environmental acquisition.

The case of the honey bee opens exciting questions on the relationships between genetics, CHC profile, and GM in the wider group of bees. In fact, honey bees, as highly eusocial bees, have colonies presenting a high genetic relatedness among workers (i.e., they essentially are all sisters) (Estoup et al. 1994) and the GM is simple and conserved, since it is acquired through frequent trophallaxis between workers (Kwong and Moran 2016). It is not necessary to be genetically related for such a similarity in both traits among workers; though in honey bees, at least the strain‐level structure of the GM seems to be partially determined by genetic relatedness (Wu et al. 2021). Hence, studying the GM and CHC profile in other bee lineages, whose social behavior differs from that of honey bees, may help to understand the relationship between genetic relatedness and the interaction between GM and CHC profile.

One of the bee taxa that can be used as a model in this sense is the family Halictidae or sweat bees. In this family, many species are solitary, but a non‐negligible number of eusocial species (around 800 out of roughly 4500 species) are known (Michener 2000; Danforth 2002; Brady et al. 2006; Schwarz et al. 2007). However, eusociality in sweat bees is far from similar to the complex, highly eusocial behavior of honey bees. Sweat bees are indeed primitively eusocial, showing a weaker and often more flexible social organization. Their colony cycle is annual, rather than perennial as in the honey bee. Queens establish their colonies in spring, during a solitary phase in which they build the initial nest structure and provision their first produced larvae, which will develop into adult workers a few weeks later (e.g., Knerer 1992). In these primitively eusocial bees, sometimes more than one founding queen is reported from a single colony (e.g., Eickwort and Eickwort 1971). Furthermore, while this phenomenon also occurs in honey bees (Free 1958), workers of halictid bees often drift among nests and can hence live part or all their life in a nest different from their natal ones, decreasing the overall colony genetic relatedness (e.g., Soro and Paxton 2009). There is evidence that such drifting behavior is favored by the generally high tolerance of workers, who are very rarely aggressive to non‐nestmates during encounters (Polidori and Borruso 2012), probably because the CHC profile‐driven nest recognition is weak (Steitz et al. 2021; Soro et al. 2011). Consequently, a remarkably variable intra‐colonial genetic relatedness has been reported in such bees (Soro and Paxton 2009; Ulrich et al. 2009). The few available studies on the GM of sweat bees revealed that it is much more complex and much less conserved than that of honey bees, being largely shaped by the environment and less by trophallaxis among workers (Mayr et al. 2021; Ronchetti et al. 2022; Shell and Rehan 2022).

Hence, the primitively eusocial sweat bees' behavioral traits are a good model to investigate the respective role of genetic relatedness and the role of the adult environment (i.e., the occupied nest) on worker‐worker similarity in CHC profile, GM, and their interaction. Here, we used as a model *Halictus scabiosae* (Rossi), which is one of the largest and most common sweat bee species in Europe. By performing a detailed analysis of genetic relatedness, CHC profile composition, and GM composition using workers collected from a number of nest aggregations, we try to answer the following questions: (1) Are the differences in CHC profile and in the GM among workers dependent on their genetic relatedness? (2) Are these traits further dependent on the adult environment (residency nest)? (3) How strongly are the CHC profile and the GM co‐varying across workers?

## 2 Materials and Methods

### 2.1 Study Species and Sample Collection

*H. scabiosae* is distributed in Central and South Europe (Gil‐Tapetado et al. 2024). This species nests in the ground, usually in compact soil with scattered vegetation, and it can be found in large aggregations (Batra 1966; Lienhard et al. 2010). *H. scabiosae* is an obligate, primitive eusocial species for which a strong tolerance between non‐nestmates and a frequent occurrence of drifting between nests were reported (Gonzalez et al. 2018; Ulrich et al. 2009). Furthermore, the workers from the first brood phase sometimes reproduce and raise a portion of the workers of the second phase (i.e., queens do not fully monopolize reproduction) (Brand and Chapuisat 2012; Ulrich et al. 2009). Both drifting and worker egg‐laying consequently lead to a flexible social organization of the colony and to an average genetic relatedness among workers lower than expected for a classical and robust matrilineal system (i.e., below 0.75) (Ulrich et al. 2009).

**FIGURE 1:**
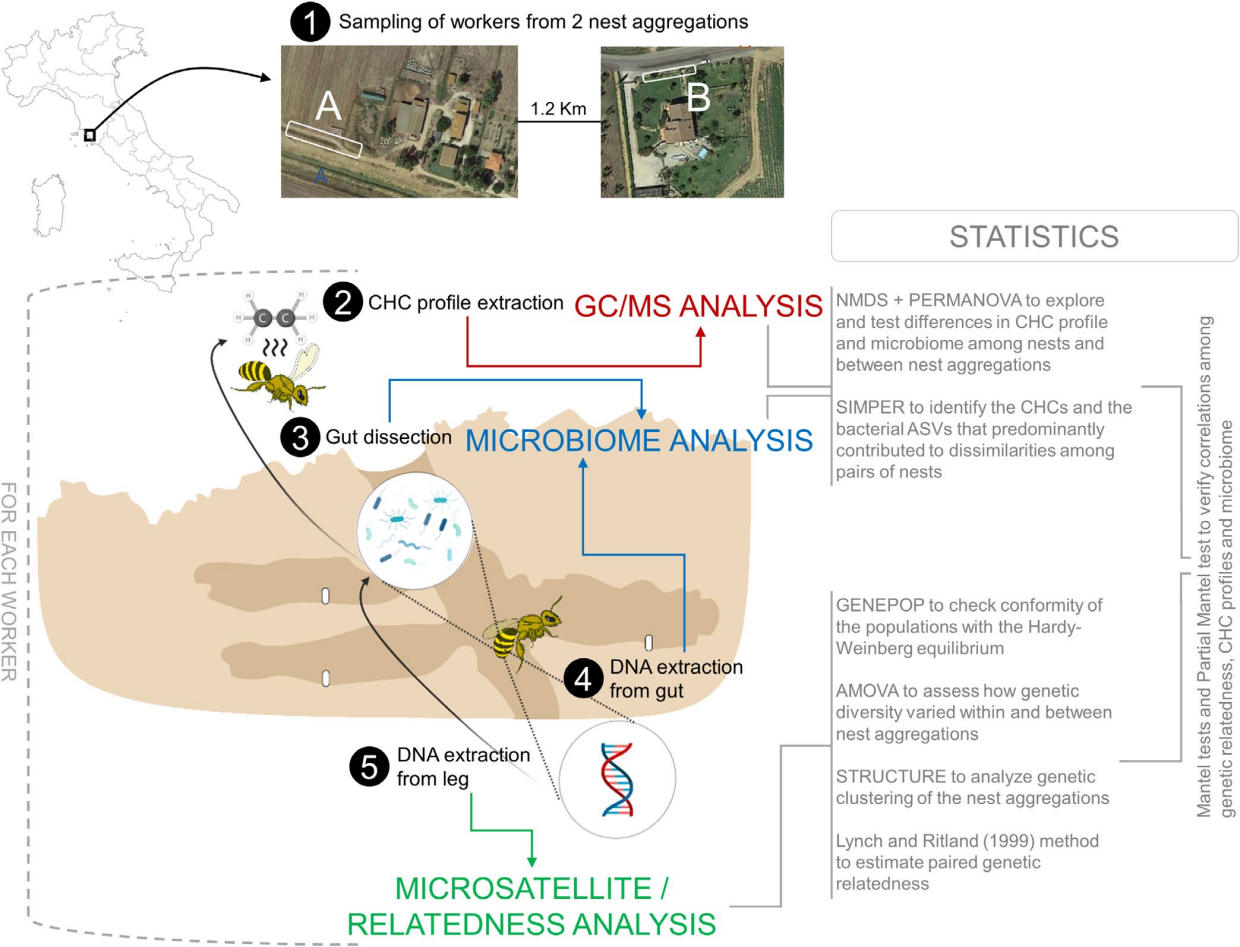
Workflow diagram linking sample collection, DNA extraction, CHC analysis, gut microbiome analysis, and statistical analyses performed on the data.

Workers of *H. scabiosae* were collected at two nest aggregations nearby the small town of Alberese, inside the Maremma Regional Park (Tuscany, Italy: 42°40′5″ N, 11°6′23″ E) (Figure S1). The two nest aggregations (hereafter: aggregation 1 and aggregation 2), composed of around 100 (aggregation 1) and 50 (aggregation 2) nests, were located at a 1.2 km distance (Figure 1, Figure S1). Aggregation 1 was found along a field path with scattered vegetation nearby the rural hotel “Agriturismo Sagrado”, while aggregation 2 was found on the walls of a small dry canal used for agricultural irrigation nearby a private home (“Casa Gialla”). The minimum distance between nest entrances at both sites was around 3–4 cm. We collected five *H. scabiosae* workers *per* nest, by netting, from a total of 18 nests (seven nests from the aggregation 1, named from A to G and 11 nests from the aggregation 2, named from H to U). To ensure nest residency, only workers about to enter with a pollen load or while exiting from the nest were collected. In total, we collected and subsequently analyzed 90 workers. Collection of the bees took place on sunny days between July 1 and July 22, 2020. The specimens were immediately stored at −20°C in a freezer at a facility available at the location of the fieldwork. After that, the sample has been brought back to the lab directly on dry ice. Subsequently, our analysis included first the extraction of the CHC profile, then the extraction of GM DNA, and finally the extraction of bee DNA, making possible the simultaneous investigation of the three aspects and their interactions across all *H. scabiosae* individuals (Figure 1 and subsections below).

### 2.2 CHC Analysis

To extract CHCs, bees were allowed to thaw and were immersed in *n*‐hexane for 10 min. Hexane treatment is not impacting the gut microbiota since hexane does not penetrate the cuticle of insects within 10 min. Hexane is indeed used to extract the chemical hydrocarbons from the cuticle of the insect while preserving the tissues under the cuticle and their genetic material, including microbiome (Mayr et al. 2021). CHC extracts were stored at −20°C and then analyzed with an Agilent 7890 gas chromatograph (GC) coupled to an Agilent 5975 Mass Selective Detector (MS) (Agilent, Waldbronn, Germany). The GC (split/splitless injector in splitless mode for 1 min, injected volume of 1 μL at 300°C injector temperature) was equipped with a DB‐5 Fused Silica capillary column (30 m × 0.25 mm ID, df = 0.25 μm, J&W Scientific, Folsom, USA). Helium was used as carrier gas with a constant flow of 1 mL/min. The GC/MS temperature program starts at 60°C with a subsequent increase of 5°C/min to 300°C and is kept isothermally at 300°C for 10 min. An ionization voltage of 70 eV (source temperature, 230°C) was set for the acquisition of the mass spectra by electron ionization (EI‐MS). The software MSD ChemStation G1701EA E.02.02.1431 was used to record and analyze the chromatograms and mass spectra. Commercially available standards, retention indices, and the detected diagnostic ions (Carlson et al. 1998) were used to identify CHC compounds.

The compounds which added less than 0.01% to the overall relative amount within each group (nest) were deleted. If there was a compound with a relative amount higher than 0.01% in a single nest, we kept it in all investigated nests for the comparative analysis. In a second step we eliminated all compounds which did not occur at least in 50% of all individuals within a group. The final matrix included 47 peaks (Table 1). Since relative peak areas of a sample are not statistically independent, we then transformed all the relative peak values following the modified Aitchison's (1986) method used by Strohm et al. (2008) (log10((% peak area/geometric mean of % peak area) + 1)), as done in previous studies (e.g., Polidori et al. 2020; Ronchetti et al. 2023). The raw chemical data are available in the Dataset S1.

### 2.3 Gut Microbiome

The gut of each specimen was extracted by dissecting the abdomen of the previously hexane‐washed individuals using flame‐sterilized forceps and scalpels under a stereomicroscope. Each gut was placed in a 1.5 mL microcentrifuge tube containing 200 μL of DNA/RNA stabilization solution (DNA/RNA Shield Lysis Tubes; Zymo Research, Irvine, CA, USA) to preserve the genetic integrity and expression profiles of samples at ambient temperatures and to completely inactivate infectious agents (viruses, bacteria, fungi, & parasites). We used sterile materials for the dissection of the gut, and we followed the extraction protocol of the manufacturer as specified. Then, the samples were frozen at −20°C and we used DNAase/RNase‐free pestles to smash the gut. Microbial gut DNA was then precipitated and purified using the ZymoBIOMICS DNA Miniprep Kit (Zymo Research, Irvine, CA, USA) and following the manufacturer's protocol for microbiome and metagenome analyses. We added two control blanks to identify possible contaminants. We sequenced one positive and four negative controls.

After acquiring the whole genomic DNA, PCR amplification of the 16S ribosomal DNA was established on the Illumina platform (Illumina 2013, 2017) for metabarcoding. The dual‐indexing strategy suggested by Kozich et al. (2013) was followed to generate a pooled amplicon library based on the V4 variable region of the gene. Hypervariable region V4 is widely used for GM analysis in Hymenoptera (Jones et al. 2018; Ashigar and Ab Majid 2020; Ramalho and Moreau 2023) and it provides a good balance between sequence length and variability, allowing for effective differentiation among closely related bacterial taxa. The V4 region is specifically designed to work well with Illumina sequencing platforms, such as the MiSeq. Targeting the V4 region allows for nearly complete overlap between paired‐end reads, which significantly reduces sequencing noise and errors. This overlap is beneficial in improving the accuracy of ASV clustering and downstream analyses, leading to more reliable interpretations of microbial diversity.

The following primers were taken to amplify the V4 region by using a dual‐indexing strategy for multiplexing: AATGATACGGCGACCACCGAGATCTACAC [8 bp‐i5 index] ATGGTAATTGTGTGCCAGCMGCCGCGGTAA, and CAAGCAGAAGACGGCATACGAGAT [8 bp‐i7 index] AGTCAGTCAGCCGGACTACHVGGGTWTCTAAT (Illumina 2016a). To reduce random effects, PCR reactions were performed in triplicates with 1 μL of template DNA in each reaction. The following thermal profile was used for amplification: denaturation at 95°C for 2 min, then amplification by using 30 cycles of 95°C for 20 s, 55°C for 15 s, and 72°C for 5 min. A final extension (72°C) of 10 min guaranteed complete amplification. After that, we combined the triplicates of each reaction and checked if the amplification was successful in a 1.5% agarose gel electrophoresis.

We normalized DNA amount between samples of each library using the Invitrogen SequalPrep Plate Normalization Kit (ThermoFisher Scientific, Life Technologies, Carlsbad, CA, USA). We used the BioAnalyzer 2200 (Agilent, Santa Clara, USA) with High Sensitivity DNA Chips to measure the fragment length distributions. The Qubit II Fluorometer and the dsDNA High‐Sensitivity Assay Kit (ThermoFisher Scientific, Life Technologies, Carlsbad, CA, USA) were used to quantify the final pool. We then loaded the library pools into 500 cycle reagent Illumina Miseq cartridges (500 cycle, v2, read length 2 × 250) along with the respective read 1 and read 2 sequencing primers. Since the MiSeq needs base diversity on every cycle, the control library for Illumina sequencing runs 5% PhiXv3 (Illumina 2016b) was loaded with the libraries. Samples were in‐house sequenced on a MiSeq platform in the Department of Human Genetics of the University of Würzburg, Germany.

For bioinformatics processing, we used VSEARCH v2.14.2 (Rognes et al. 2016) according to the pipeline available at https://github.com/chiras/metabarcoding_pipeline (Leonhardt et al. 2022). First, FASTQ forward and reverse sequences were joined (maximum sequence differences of 10 base pairs of each sequence). We used the Unoise3 algorithm (Edgar 2016a) to denoise sequences and dereplicate into amplicon sequence variants (ASVs) after quality filtering (max EE < 1.0, read length > 250 and < 300 bp), dereplication, singleton exclusion, and chimera removal. Then, with the RDP v16 reference database (Cole et al. 2014) we taxonomically classified ASVs first by direct global alignments and a threshold of > 7% identity, and then for remaining unclassified reads hierarchically with the SINTAX (Edgar 2016b) implementation of VSEARCH as deep as possible with a threshold of 0.8. Further data analysis was conducted in R v3.5.2 (R Foundation, Vienna, Austria) using the package “phyloseq” (McMurdie and Holmes 2013). We filtered from the dataset the ASVs that were assigned as chloroplasts or mitochondria, and the 10 most abundant ASVs that were present in the negative controls. Prior to statistical analysis, we transformed read counts to relative (%) abundances of gut ASVs for each individual and removed ASVs representing less than 1% abundance within each sample. The raw microbiome data are available in the Dataset S1.

### 2.4 DNA Extraction and Genotype Determination

We studied genetic relatedness between workers using microsatellite markers. DNA extraction was performed from one leg of each individual using Chelex 100 (Walsh et al. 1991). The Chelex extraction method has become a staple in the field of honey bee research due to its rapidity and efficacy in DNA extraction (Evans et al. 2013). This method has been demonstrated to yield DNA of sufficient quality for microsatellite analysis. Consequently, it was selected as the primary extraction method for the samples in this study, and its effectiveness was subsequently validated. Eight microsatellite markers were used in this study, with primers reported in Table S1. Two different multiplex reactions were designed (Multiplex 1: LHMS10, rub73, rub02, rub72; Multiplex 2: rub37b, rub30, rub77, LM27) using forward primers fluorescent‐labeled and amplified under the following PCR conditions: initial denaturation at 94°C for 3 min; 35 cycles of 30 s at 94°C, 30 s at 55°C, and 45 s at 64°C; a final elongation for 10 min at 64°C. In order to ascertain the feasibility of the multiplex reactions, a polymerase chain reaction (PCR) test was performed. This involved the amplification of five samples of *H. scabiosae* , both individually and in combination in the different multiplexes. The annealing temperature was selected based on the articles in which the primers were designed (Kukuk et al. 2002; Paxton et al. 2003; Soro and Paxton 2009). Once the efficacy of the multiplex reaction had been verified, the remaining samples were amplified.

Amplified products were sent for detection to the company Secugen S.L (Madrid, Spain), and the identification of alleles was carried out using GENEMAPPER 3.7 software (Applied Biosystems) to export the data matrix for subsequent analysis.

### 2.5 Statistical Analyses

The number of microsatellite total and private alleles of each nest aggregation, as well as the unbiased expected heterozygosity (uH_e_) were obtained using GenAlEx 6.5 (Peakall and Smouse 2006). The presence of null alleles was evaluated with Micro‐Checker version 2.2.3 (Van Oosterhout et al. 2004), and the conformity of the populations with the Hardy–Weinberg equilibrium was checked using GENEPOP on the web version 4.1 (Rousset 2008). Analysis of molecular variance (AMOVA) was carried out based on 999 replicates using GeneAlex 6.5 (Peakall and Smouse 2006) to assess how genetic diversity varied within and between nest aggregations. Paired relatedness between workers, estimated in terms of probabilities of identity by descent, was calculated following Lynch and Ritland (1999) with the software COANCESTRY (Wang 2011). To perform this analysis, one of the loci had to be eliminated because it was monomorphic; furthermore, five individuals (three from aggregation 1 and two from aggregation 2) were eliminated because they amplified for only three polymorphic loci. The genetic relatedness data are available in the Dataset S1. Finally, in order to analyze the genetic clustering of the *H. scabiosae* aggregations, the STRUCTURE v2.3.4 program (Pritchard et al. 2000) was used. All possible existing populations (K; 1–10) (burn‐in: 1 × 104; MCMC: 105) were simulated using 10 iterations each to obtain the most probable one according to the ∆*K* value (Evanno et al. 2005) obtained with STRUCTURE HARVESTER web v0. 6.94 (Earl and von Holdt 2012).

To answer the question of whether nestmates possess a more similar CHC profile and more similar GM, we first explored the overall CHC profile and GM of the workers with accumulated bar charts on hydrocarbon class level and bacterial class level. Then, we tested for both chemical and microbiome differences between nests by performing multivariate analyses, based on Bray–Curtis dissimilarity matrices, which are suitable for zero‐inflated datasets (Leyer and Wesche 2007). These analyses do not require a priori grouping of individuals, meaning that these methods allow pattern formation that is exclusively based on CHC or GM dissimilarities. In particular, we used nonmetric multidimensional scaling (NMDS), which is a non‐parametric method that does not assume linearity among variables (McCune and Grace 2002). The resulting plots show the spatial distances between individuals, that is, their CHC or GM distances. In the NMDS, deviations are expressed in terms of “stress,” for which values ≤ 0.15 indicate a good fit of ordination (Kruskal 1969). PERMANOVA (Non‐Parametric MANOVA) was employed to test for differences among nests and between nest aggregations, based on 9999 permutations. Similarity percentages (SIMPER) were calculated to identify the CHCs and bacterial ASVs that predominantly contributed to the Bray‐Curtis dissimilarities among pairs of nests (Clarke 1993).

Finally, we tested for correlations among genetic relatedness, CHC profiles, and GM using a series of Mantel tests (Mantel 1967). This approach tests association between distance matrices and is permutation‐based to counter dependency issues in dissimilarity matrix correlations. It was applied to verify if (1) genetically more closely related workers possess a more similar CHC profile, (2) genetically more closely related workers possess a more similar GM, and (3) workers with a more similar CHC profile have a more similar GM. We also performed an additional partial Mantel test simultaneously based on all three matrices (Smouse et al. 1986). In this case, *r* represents the partial correlation coefficient of CHC and MG given genetic relatedness, that is, controlling for the latter variable. All these tests are based on Pearson correlation coefficients, with 9999 permutations. The statistical analyses were performed in PAST 3.04 (Paleontological Statistics Software Package) (Hammer and Harper 2001) and in R v 3.5.0 (R Core Team 2021), through RStudio v 1.1.453 (R Studio Team 2015).

## 3 Results

### 3.1 Genetic Analysis

**FIGURE 2:**
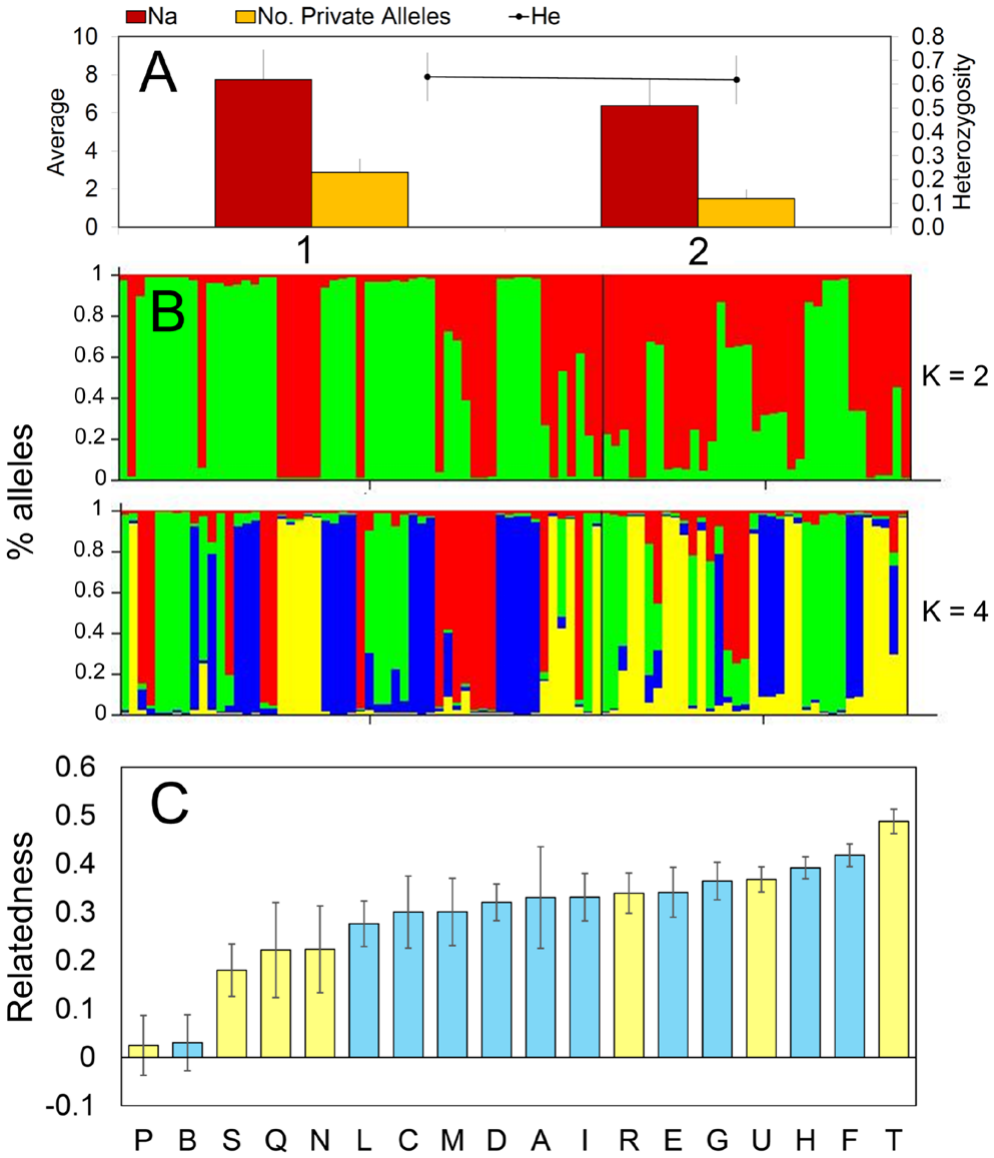
(A) Mean values of total number of alleles (N), privative alleles (No. Private Alleles) and expected heterozygosity (He) for each of the two nest aggregations. (B) Clusters for both *K* = 2 and *K* = 4 (*K* = number of genotypic clusters), inferred with Structure v2.3.4 software. The cluster membership of each individual is shown by the color composition of the vertical lines, with the length of each colored part of the line being proportional to the estimated membership coefficient. (C) Mean worker‐worker genetic relatedness (±SE), in increasing order, within the nests (blue: Nest aggregation 1, yellow: Nest aggregation 2).

All the microsatellite markers presented allele polymorphism among all samples except for LM27, which showed the presence of a single allele in all individuals from both aggregations. Thus, the number of alleles per locus varied from 1 to 14 (Table S1), with a mean of 7.063 ± 1.556 alleles over loci detected across aggregations. No evidence for null alleles was found for any loci and in any aggregation. Both nest aggregations presented similar values of total and private alleles (Table 1, Figure 2A), although slightly higher in aggregation 2 than in aggregation 1. Unbiased expected heterozygosity (uH_e_) values were similar in both aggregations (0.637 ± 0.102 and 0.629 ± 0.102). The inbreeding coefficient (*F*_is_) showed negative values for every locus, and the results of the Hardy–Weinberg equilibrium analysis showed a clear departure from the assumed proportions in both aggregations separately and as a whole (Table 1, Figure 2A). AMOVA analysis showed that the molecular variance was greater among the individuals occurring within each aggregation, and only 2% of the total was due to the differences between aggregations (*p* = 0.011).

**TABLE 1:** Alleles and heterozygosity data for the two nest aggregations of *H. scabiosae* .

Nest aggregation	Na	No. Private Alleles	Ho	He	HW (*p*)
A					
Mean	6.375	1.5	0.686	0.619	< 0.0001
SE	1.362	0.463	0.105	0.101	
B					
Mean	7.75	2.875	0.702	0.631	< 0.0001
SE	1.556	0.718	0.114	0.101	

Grand Mean and SE over Loci and nest aggregation				
	Na	Ho	He	HW (*p*)
Total				
Mean	7.063	0.694	0.625	< 0.0001
SE	1.014	0.075	0.069	

Genetic differentiation (*F*_st_) between aggregations was 0.010 ± 0.002. Clustering analyses obtained with Structure Harvester assigned the highest value of ∆*K* to *K* = 2, suggesting this aggregation structure as the most likely (although due to the limitations of the method, the program is not able to indicate the possibility of *K* = 1 as the most likely). Structure analysis did not show a clear differentiation between the two nest aggregations for the *K* = 2 simulation (Figure 2B). However, it showed a certain difference in the allocation of individuals to each of the two simulated groups, with one being more abundant in one aggregation, and vice versa. As the value of *K* is increased (e.g., *K* = 4), differences among nests also appeared, but with a less clear correspondence with the two nest aggregations. However, the simulation of so many well‐defined groups could indicate and corroborate a greater difference among individuals within the aggregation than between aggregations.

Average paired genetic relatedness greatly varied across nests but was never high, with five nests (P, B, S, Q, N) showing very low relatedness (0.01–0.17) and three nests (H, F, T) showing moderate values (0.31–0.39) (Figure 2C). The remaining nests presented values between 0.20 and 0.29 (Figure 2C).

### 3.2 CHC Profiles

**FIGURE 3:**
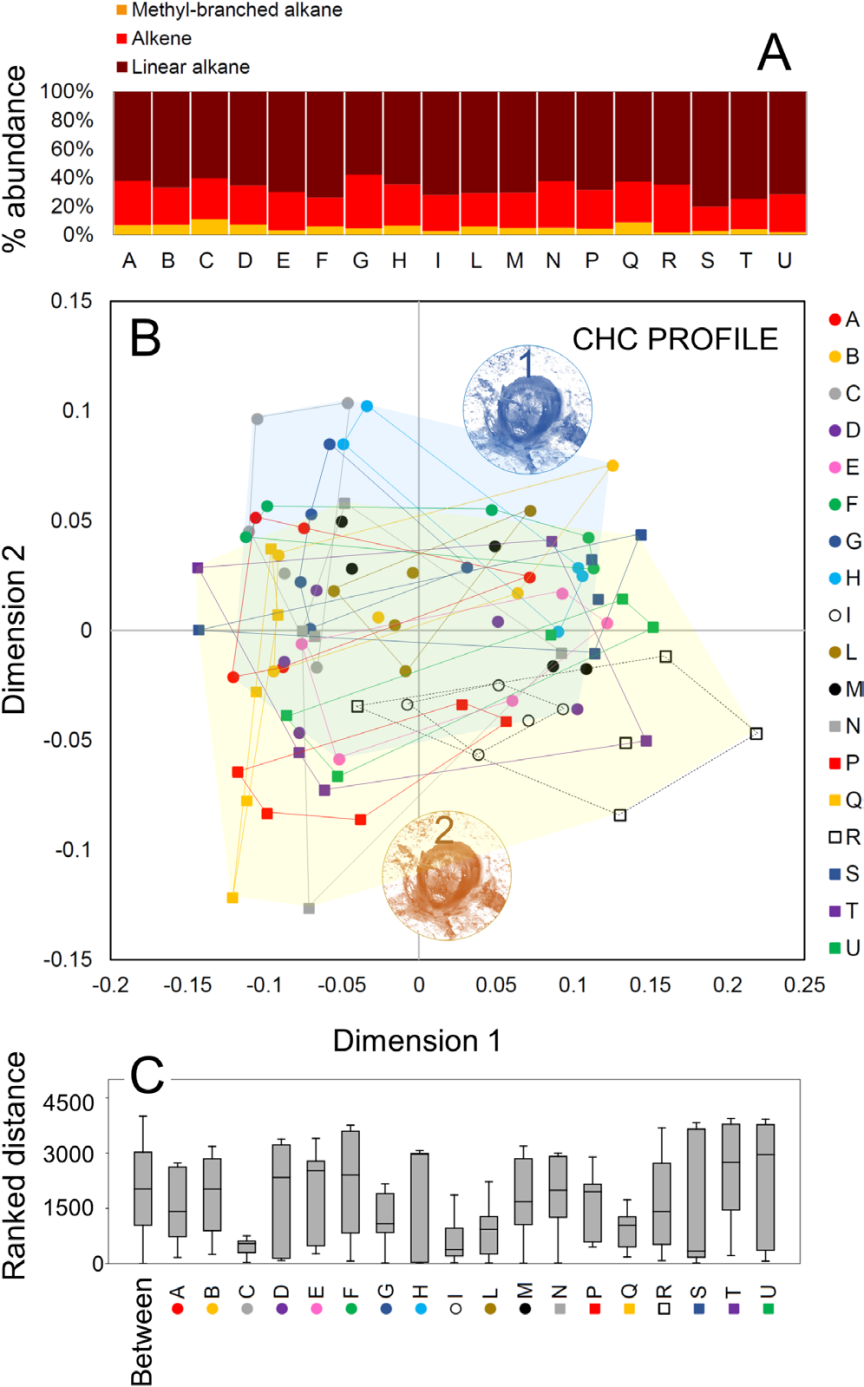
(A) Relative abundance of the three CHCs classes found on the cuticle of *H. scabiosae* , with each bar identifying a nest. (B) Nonmetric multidimensional scaling (NMDS) based on Bray–Curtis distances of all individual CHC profiles from the studied nests. Letters identified nests, circles identified nest aggregation 1, squares identified nest aggregation 2; blue transparent area includes all nests from aggregation 1, yellow transparent area includes all nests from aggregation 2. (C) Box‐and‐whisker plots showing medians (horizontal lines within boxes), 1° and 3° quartile (horizontal lines closing the boxes), and maximum and minimum values (ends of the whiskers) for the Bray‐Curtis distances within each studied nest, and between nests.

The CHC profiles of *H. scabiosae* workers included overall 47 compounds, which covered three hydrocarbon classes: *n‐*alkanes, monomethyl‐branched alkanes, and alkenes (Table 2). All classes of compounds were represented in all individuals. Overall, we found a similar relative abundance of the three classes of hydrocarbons in both aggregations (Figure 3A). The *n‐*alkanes were the most abundant compounds in both aggregation 1 (Mean + SE = 62.6% ± 0.3) and 2 (65.3% ± 0.5), followed by alkenes (aggregation 1: 30.9% ± 0.2; aggregation 2: 30.2% ± 0.3) (Figure 3A). Monomethyl‐branched alkanes were less abundant, ranging from 6.5% ± 0.1 in aggregation 1 to 5.1% ± 0.1 in aggregation 2 (Figure 3A). Compounds spanned a carbon chain length between 19°C and 33°C atoms.

**TABLE 2:** Mean ± standard error for the relative abundance of all hydrocarbons occurring on the cuticle of *H. scabiosae* at the two studied nest aggregations.

Compound	Aggregation A	Aggregation B
C19	0.00	0.16 ± 0.12
C20	0.24 ± 0.05	0.27 ± 0.05
C21	18.30 ± 2.14	22.24 ± 3.21
C22	0.62 ± 0.08	0.54 ± 0.09
C23en	0.32 ± 0.08	0.01 ± 0.01
C23	3.29 ± 0.19	3.39 ± 0.29
9‐MeC23	0.19 ± 0.04	0.10 ± 0.02
7‐MeC23	0.19 ± 0.03	0.09 ± 0.02
5‐MeC23	0.11 ± 0.03	0.06 ± 0.01
3‐MeC23	0.21 ± 0.12	0.13 ± 0.06
C24	0.23 ± 0.03	0.16 ± 0.02
C25en:1	0.51 ± 0.06	0.37 ± 0.07
C25en:2	0.76 ± 0.08	0.57 ± 0.09
C25	2.32 ± 0.11	2.64 ± 0.16
5‐MeC25	0.60 ± 0.05	0.38 ± 0.03
C26en	0.03 ± 0.02	0.00
C26	0.48 ± 0.03	0.47 ± 0.03
C27en:1/C27dien	0.10 ± 0.02	0.10 ± 0.04
C27en:2	1.02 ± 0.25	3.27 ± 1.02
C27en:3	2.18 ± 0.29	2.96 ± 0.43
C27en:4	1.44 ± 0.12	1.23 ± 0.18
C27	10.12 ± 0.49	11.76 ± 0.94
13‐;11‐MeC27	0.99 ± 0.06	0.89 ± 0.10
7‐MeC27	0.18 ± 0.01	0.14 ± 0.02
5‐MeC27	0.23 ± 0.04	0.15 ± 0.03
C28en:1	0.01 ± 0.01	0.00
C28en:2	1.03 ± 0.49	0.03 ± 0.02
C28	0.80 ± 0.03	0.75 ± 0.04
C29en:1	2.16 ± 0.19	2.29 ± 0.43
C29en:2	6.89 ± 0.39	8.23 ± 0.90
C29en:3	4.48 ± 0.31	4.72 ± 0.50
C29en:4	2.47 ± 0.21	1.33 ± 0.24
C29	13.17 ± 0.66	13.05 ± 1.30
15‐;13‐;11‐MeC29	0.94 ± 0.06	0.75 ± 0.08
9‐MeC29	0.38 ± 0.05	0.27 ± 0.04
7‐MeC29	1.12 ± 0.11	0.69 ± 0.11
5‐MeC29	0.36 ± 0.02	0.22 ± 0.03
C30	1.25 ± 0.07	1.15 ± 0.08
C31en:1	5.92 ± 0.50	3.31 ± 0.44
C31en:2	1.12 ± 0.12	0.95 ± 0.18
C31en:3	0.52 ± 0.11	0.83 ± 0.41
C31	11.68 ± 0.63	8.70 ± 0.93
15‐;13‐;11‐MeC31	0.41 ± 0.05	0.21 ± 0.07
9‐MeC31	0.27 ± 0.05	0.29 ± 0.16
5‐MeC31	0.11 ± 0.05	0.09 ± 0.06
3‐MeC31	0.18 ± 0.11	0.02 ± 0.02
C33	0.10 ± 0.06	0.00

Although not well visible in NMDS (stress = 0.14), the PERMANOVA indicated a significant separation between nests (total sum of square = 6.9, within‐group sum of square = 4.0, *F* = 3.2, *p* = 0.0001) (Figure 3B) and between nest aggregations (total sum of square = 6.63, within‐group sum of square = 6.38, *F* = 3.33, *p* = 0.03). Hence, there is a tendency for all members from a nest to at least partially share their CHC profile. However, the NMDS inter‐worker variability in their CHC profile among nests was large. Workers from some nests, such as nest S and nest U, had overall a highly variable CHC profile overlapping with that of many non‐nestmates, while workers from other nests, such as nest C and nest I have a more coherent profile and differ from non‐nestmate workers (Figure 3B,C).

The SIMPER analysis showed that four *n‐*alkanes, and in particular C21, mostly accounted for the dissimilarity among the studied nests (Figure S2). Also, a number of alkenes contributed to the dissimilarity among nests, while methyl‐branched alkanes seemed to have a low impact (Figure S2).

### 3.3 Gut Microbiome

**FIGURE 4:**
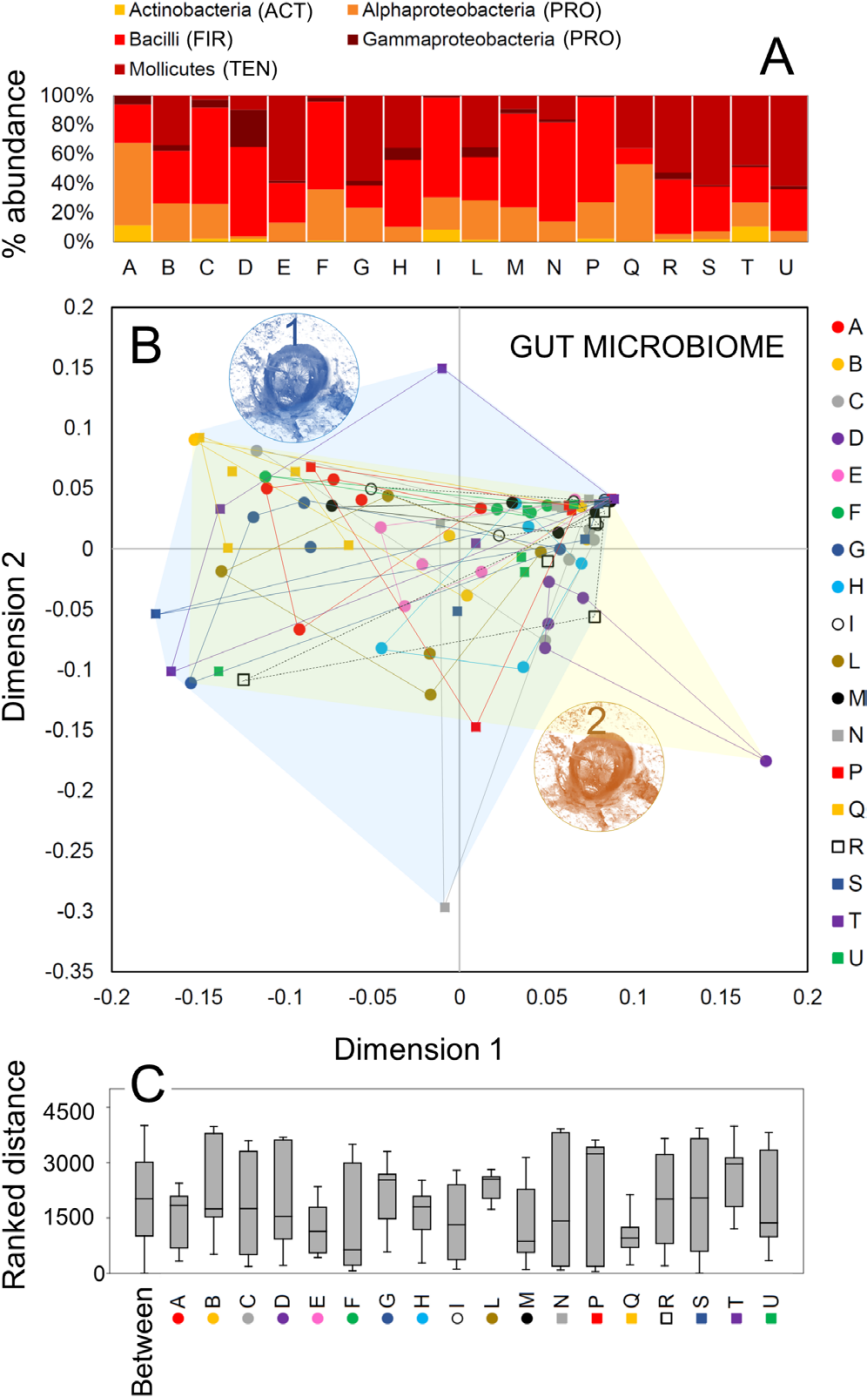
(A) Relative abundance of the five bacterial classes found in the GM of *H. scabiosae* , with each bar identifying a nest. For each class, the bacterial phylum is in brackets (ACT, Actinobacteria; FIR, Firmicutes; PRO, Proteobacteria; TEN, Tenericutes). (B) Non‐metric multidimensional scaling (NMDS) based on Bray–Curtis distances of all individual GM from the studied nests. Letters identified nests, circles identified nest aggregation 1, squares identified nest aggregation 2; blue transparent area includes all nests from aggregation 1, yellow transparent area includes all nests from aggregation 2. (C) Box‐and‐whisker plots showing medians (horizontal lines within boxes), 1° and 3° quartile (horizontal lines closing the boxes), and maximum and minimum values (ends of the whiskers) for the Bray–Curtis distances within each studied nest, and between nests.

Sequencing resulted in 2,629,405 quality filter reads and an average number of 29,216 reads per sample (i.e., worker). A total of 24 ASVs were detected overall in the sample, belonging to four phyla. Firmicutes, Proteobacteria, and Tenericutes were the most abundant phyla, followed by Actinobacteria (Figure 4A). Within Firmicutes, Bacilli was the most abundant class, and it also represented one of the most abundant classes overall across samples. However, Alphaproteobacteria (Proteobacteria) and Mollicutes (Tenericutes) were also very abundant classes and, in certain samples, more abundant than Firmicutes (Figure 4A). The most abundant genus of Firmicutes was *Apilactobacillus* (50.06% ± 0.06), followed by *Lactococcus* (5.38% ± 0.16) (Table 3). Dominant Proteobacteria were *Saccharibacter* (19.68% ± 2.22) and *Acinetobacter* (7.51% ± 4.77) (Table 3). *Spiroplasma* was the sole genus of Tenericutes occurring in the whole dataset (9.40% ± 4.33) (Table 3). Overall, five genera (*Acinetobacter*, *Saccharibacter*, *Apilactobacillus*, *Lactococcus*, and *Spiroplasma*) occurred in at least 30% of all bees (up to 89%) with an average abundance, in the individuals where they occur, of at least 10% (up to 50%) and together can be considered the core microbiome of *H. scabiosae* (Table S2).

**TABLE 3:** Mean ± standard error for the % abundance of all the ASVs composing the gut microbiome of *H. scabiosae* at the two studied nest aggregations. Taxonomic classification of the ASVs is also shown.

Phylum	Class	Order	Family	ASV	Aggregation A	Aggregation B
Actinobacteria	Actinobacteria	Mycobacteriales	Nocardiaceae	*Rhodococcus*	0.94 ± 0.36	1.21 ± 0.46
				*Mycobacteriales* spp.	0.00	0.05 ± 0.05
Firmicutes	Bacilli	Bacillales	Staphylococcaceae	*Staphylococcus*	0.00	0.02 ± 0.02
		Lactobacillales	Lactobacillaceae	*Apilactobacillus*	50.02 ± 3.75	52.80 ± 4.35
				*Fructobacillus*	0.00	0.59 ± 0.48
				*Weissella*	0.27 ± 0.27	1.33 ± 1.33
			Streptococcaceae	*Lactococcus*	5.50 ± 1.61	5.44 ± 1.83
				*Lactobacillales* spp.	0.27 ± 0.10	0.07 ± 0.04
Proteobacteria	Alphaproteobacteria	Rhodobacterales	Rhodobacteraceae	*Paracoccus*	0.00	0.04 ± 0.04
		Rhodospirillales	Acetobacteraceae	*Gluconobacter*	0.08 ± 0.04	0.38 ± 0.15
				*Saccharibacter*	21.25 ± 3.23	17.83 ± 3.06
		Rickettsiales	Rickettsiaceae	*Wolbachia*	0.16 ± 0.09	0.70 ± 0.32
	Gammaproteobacteria	Enterobacterales	Enterobacteriaceae	*Cronobacter*	0.00	0.03 ± 0.03
	Gammaproteobacteria			*Izhakiella*	0.02 ± 0.02	0.00
				*Enterobacteriaceae* spp.	0.00	0.02 ± 0.02
			Erwiniaceae	*Pantoea*	0.00	0.12 ± 0.06
			Morganellaceae	*Arsenophonus*	2.61 ± 1.81	0.98 ± 0.86
			Pectobacteriaceae	*Sodalis*	0.02 ± 0.02	0.15 ± 0.12
			Yersiniaceae	*Serratia*	0.00	0.10 ± 0.07
				*Enterobacterales* spp.	0.03 ± 0.03	0.12 ± 0.05
		Pseudomonadales	Moraxellaceae	*Acinetobacter*	10.88 ± 1.83	6.13 ± 1.46
				*Enhydrobacter*	0.00	0.05 ± 0.05
				*Gammaproteobacteria* spp.	0.00	0.07 ± 0.07
Tenericutes	Mollicutes	Entomoplasmatales	Spiroplasmataceae	*Spiroplasma*	6.34 ± 1.93	9.62 ± 2.80

Although not well visible in the NMDS (stress = 0.15), the PERMANOVA indicated a significant separation between nests (Figure 4B) (total sum of square = 15.88, within‐group sum of square = 11.32, *F* = 1.7, *p* = 0.004) but not between nest aggregations (total sum of square = 14.88, within‐group sum of square = 14.55, *F* = 1.85, *p* = 0.12). The GM was variable not only among nests but also within nests, and intra‐nest variance was highly variable, with workers from some nests, such as nest T and P, having a very variable GM, while workers from other nests, such as Q and E, have a more conserved GM and differing from the GM of non‐nestmate workers (Figure 3B,C).

The SIMPER analysis showed that the relative abundance of *Apilactobacillus* and *Saccharibacter*, the most abundant ASVs in the samples, had the strongest contribution to GM dissimilarity between the studied nests (Figure S2). Other relevant ASVs contributing to the GM dissimilarity were *Spiroplasma*, *Acinetobacter*, and *Lactococcus*.

### 3.4 Mantel Test: Genetic Relatedness vs. CHC Profile and Gut Microbiome

There was a significant correlation between the genetic relatedness and the GM similarity (*r* = 0.04, *p* = 0.007) and between the genetic relatedness and the CHC profile similarity (*r* = 0.07, *p* < 0.001). Distances in GM and in CHC profiles were highly and positively correlated across workers (*r* = 0.18, *p* < 0.0001), even after controlling for genetic relatedness (Partial Mantel test, *r* = 0.17, *p* < 0.0001).

## 4 Discussion

Our findings confirm a high variation of genetic relatedness between workers of *H. scabiosae* within nest aggregations, sometimes even within colonies, as it was observed in other populations of this bee species (Ulrich et al. 2009; Brand and Chapuisat 2012; Gonzalez et al. 2018). Workers may have drifted between nest aggregations that are separated by roughly 1 km, that is, a distance according to the maximum foraging distance expected for a bee species with this body size (Zurbuchen et al. 2010). Ulrich et al. (2009) reported that drifting behavior seems to be common among nests of this species, and these drifters are not closely related to the members of the occupied nests. This means that drifting might occur to decrease reproductive competition among related females (Ulrich et al. 2009). Similar behavior has been reported in other eusocial Halictidae (Kukuk et al. 2005) and might be explained by similar reasons overall in primitively eusocial sweat bees. Thus, ecological constraints can determine group formation even beyond kinship, as it was also recently demonstrated in the facultative eusocial bee *Xylocopa sonorina* (Ostwald et al. 2021). Not only workers, but also queens at the nest‐founding phase can drift, abandoning their nests after having been usurped by conspecific, nest‐less queens (Brand and Chapuisat 2016).

The CHC profiles of *H. scabiosae* workers showed a strong intra‐nest variability, despite overall nest membership accounting for CHC similarity. Indeed, the PERMANOVA depicted significant separation of the individuals by nests. *H. scabiosae* is a primitively eusocial bee with guards at the nest entrances to avoid incursions of natural enemies. A relatively weak—though appreciable—nest‐dependent CHC profile might contribute to the increasing of tolerance of non‐nestmates by reducing aggression (Gonzalez et al. 2018). Since drifting between nests, worker reproduction, and multiple foundresses lead to a reduced intra‐colony relatedness (which is correlated with high levels of tolerance among nestmates, Polidori and Borruso 2012; Gonzalez et al. 2018), it is not surprising to find a partial overlap of the chemical profiles between some of the nests. In this way, drifters may have more chances to infiltrate into a non‐natal nest, a phenomenon that was also observed in workers of the ant *Formica fusca* L. (Formicidae) (Caliari Oliveira et al. 2022). Moreover, Caliari Oliveira et al. (2022) showed that the environment is a more important factor than genetic relatedness in shaping the CHC profile of this ant species. Given this, genetic relatedness also seems to explain at least partially CHC profile similarity among workers. Hence, this trait has probably an heritable component, as it was seen in other social, as well as solitary, insects (e.g., Dronnet et al. 2006; Thomas and Simmons 2008). The CHC profile in *H. scabiosae* may thus have a genetic component, but it could be significantly altered in adult workers during nest digging and contacts between nestmates in the occupied nest.

For the GM, genetic relatedness did correlate with the community of gut symbionts of the host, while current nest residency also seems to account for GM similarity, as PERMANOVA showed. Hence, we can hypothesize that the natal nest environment would thus largely shape the GM in *H. scabiosae* , since all sisters probably share a similar bacterial environment, both from shared food (pollen) resources and shared nests. It is thus possible that the GM composition is not explained by genetic relatedness itself, but by the shared conditions during development. However, further bacteria may be acquired from the occupied nests as adults, leading to a partial role of nest sharing as a source of microbiome similarity among workers at their adult stage.

In honey bees it has been shown that genetic relatedness can shape the strain‐level structure of the GM (Wu et al. 2021). However, the highly eusocial honey bees represent a unique case among bees: newly born individuals (after pupation), which possess a sterilized gut, acquire gut symbionts mainly through social contacts with adult siblings (Martinson et al. 2012). In contrast, wild bees, which also show essentially no GM right after emerging from the pupae (Voulgari‐Kokota, McFrederick, et al. 2019), inoculate their gut very likely from the nest environment and from visited flowers (e.g., Keller et al. 2021). However, whether the nest environment is a hub to vertically transmit microbes is not known. We argue that the first days of the life of adult bees determine to a large part their GM, which could then likely change very slowly. However, very recent investigations suggest that even solitary species as the carpenter bee genus *Xylocopa* (Hymenoptera: Apidae), which often occur in shared nests without forming eusocial colonies, show a specialized, host‐restricted GM (Holley et al. 2022) that can be partially transmitted by nestmates in such a shared nests. Even some megachilid bees have a host‐specific GM (Voulgari‐Kokota, Grimmer, et al. 2019). We argue that the GM of *H. scabiosae* can be partially acquired from the nest environment as shown in other bee species (e.g., fecal remains, pollen remains, and soil bacteria) (e.g., *Ceratina* spp., Shell and Rehan 2022; *Megachilidae* spp., Voulgari‐Kokota, Ankenbrand, et al. 2019). This acquisition occurs first during the emergence of the adult from the natal nest (e.g., Keller et al. 2021) (resulting in the observed effect of genetic relatedness) and then during activity in the residency nest (leading to similarity between workers from the same occupied nest).

Finally, the strongest co‐variation in our study was between CHC profile similarity and GM similarity across workers, even after controlling for their genetic relatedness, indicating an interaction between these factors that may be independent of nest origin or current environment, which may lead to potential transitional states of the microbiome. In *D. melanogaster* , it has been shown that genetic relatedness had a strong influence on microbiome diversity, which in turn also significantly covaried with the CHC profiles (García‐Roa et al. 2022). This seems to resemble what we have found in *H. scabiosae* since genetic relatedness plays a role in CHC profile similarity. However, our results also suggest, as in *Drosophila*, an influence of the GM on the CHC profile. In honey bees, gut symbionts support nestmate recognition by affecting the CHC profile (Vernier et al. 2020). However, a causal effect of the GM on the CHC profile in *H. scabiosae* has still to be proven. For example, the disruption of the GM with antibiotics may shed some light on the covariation with the CHC profile. Few studies so far showed an association between certain bacteria and CHC profiles, and these studies mostly focused on the maternally inherited intracellular *Wolbachia* alpha‐proteobacteria and less on the GMs. *Wolbachia*‐infected males of both *Drosophila paulistorum* Dobzhansky & Pavan (Diptera) and *Armadillidium vulgare* (Latreille) (Isopoda) showed different compositions of sex pheromones and CHC profiles, respectively, compared with uninfected individuals (Schneider et al. 2019; Richard 2017). Additionally, *Wolbachia*‐infected females of the wasp *Myrmilla capitata* (Lucas) (Mutillidae) have a different CHC profile compared with uninfected females (Ronchetti et al. 2023). There is evidence that other endosymbionts affect insect host CHC profiles (Engl et al. 2018; Hertaeg et al. 2021), but so far, the role of gut bacteria in shaping the bouquet of odors in Hymenoptera remains largely unexplored.

In conclusion, our results suggest that there is an acquisition of gut bacteria right after metamorphosis in the natal nest and a subsequent conformation upon joining a new nest. On the other hand, our results show an effect of genetic relatedness on the CHC profile, which may then be further changed by contacts between workers and nest soil in the occupied nests as adults, as has been shown in other Hymenoptera (Singer and Espelie 1996; Breed et al. 1988). Further studies may shed light on the causal relationship between microbiomes and CHC profiles in this primitively eusocial bee.

## Author Contributions

**Federico Ronchetti:** conceptualization (equal), data curation (equal), formal analysis (equal), investigation (equal), methodology (equal), writing – original draft (equal), writing – review and editing (equal). **Thomas Schmitt:** conceptualization (equal), data curation (equal), formal analysis (equal), funding acquisition (equal), investigation (equal), methodology (equal), resources (equal), supervision (equal), writing – review and editing (equal). **Alexander Keller:** conceptualization (equal), data curation (equal), formal analysis (equal), funding acquisition (equal), investigation (equal), methodology (equal), resources (equal), supervision (equal), writing – review and editing (equal). **Andrés García‐Reina:** formal analysis (equal), investigation (equal), methodology (equal), writing – review and editing (equal). **Pilar De la Rua:** formal analysis (equal), methodology (equal), writing – review and editing (equal). **Ingolf Steffan‐Dewenter:** conceptualization (equal), funding acquisition (equal), writing – review and editing (equal). **Carlo Polidori:** conceptualization (equal), formal analysis (equal), funding acquisition (equal), investigation (equal), resources (equal), supervision (equal), writing – original draft (equal), writing – review and editing (equal).

## Conflicts of Interest

The authors declare no conflicts of interest.

## Supporting information


**Dataset S1.** Raw data for CHC profile, GM bacterial composition and genetic relatedness.

**Figure S1.** Location of the two nest aggregations of *H. scabiosae* (picture of the areas retrieved from Googlemap).

**Figure S2.** SIMPER contributions to nest‐nest dissimilarity of (A) the 10 most important CHC compounds and (B) the 10 most important bacterial ASVs.

**Table S1.** The eight microsatellite markers and primers used in this study.

**Table S2.** The five genera of bacteria likely representing the core microbiome of *H. scabiosae* .

## Data Availability

Raw microbiome sequencing data have been uploaded to the SRA at NCBI with the Accession number PRJNA1253914 and are available at: http://www.ncbi.nlm.nih.gov/bioproject/1253914, and the final microbiome dataset is provided in the Dataset S1. Bee genotype data have been uploaded to Dryad and are available at https://doi.org/10.5061/dryad.7wm37pw0w, and the final dataset of genetic relatedness is provided in the Dataset S1. Chemical data are provided in the Dataset S1.
