# Supplementary figures and images for: The Significance of Genetic Relatedness and Nest Sharing on the Worker‐Worker Similarity of Gut Bacterial Microbiome and Cuticular Hydrocarbon Profile in a Sweat Bee
Source: Ecol Evol. 2025 Jun 9;15(6):e71519. doi: 10.1002/ece3.71519 (PMC12146657; doi:10.1002/ece3.71519)

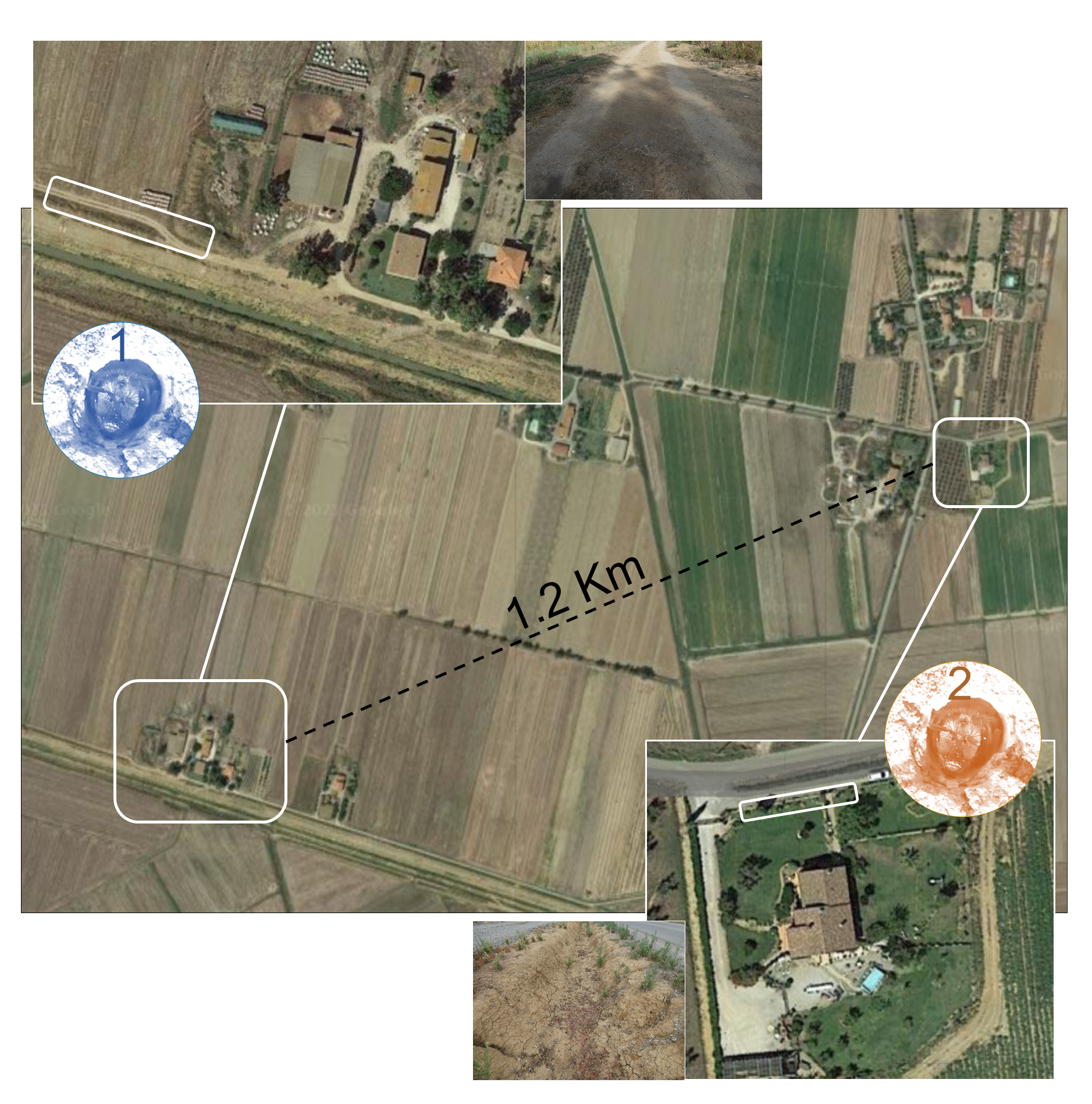

Supplement: Supplementary file 2 — Figure S1. Location of the two nest aggregations of H. scabiosae (picture of the areas retrieved from Googlemap). [file ECE3-15-e71519-s004.tif]

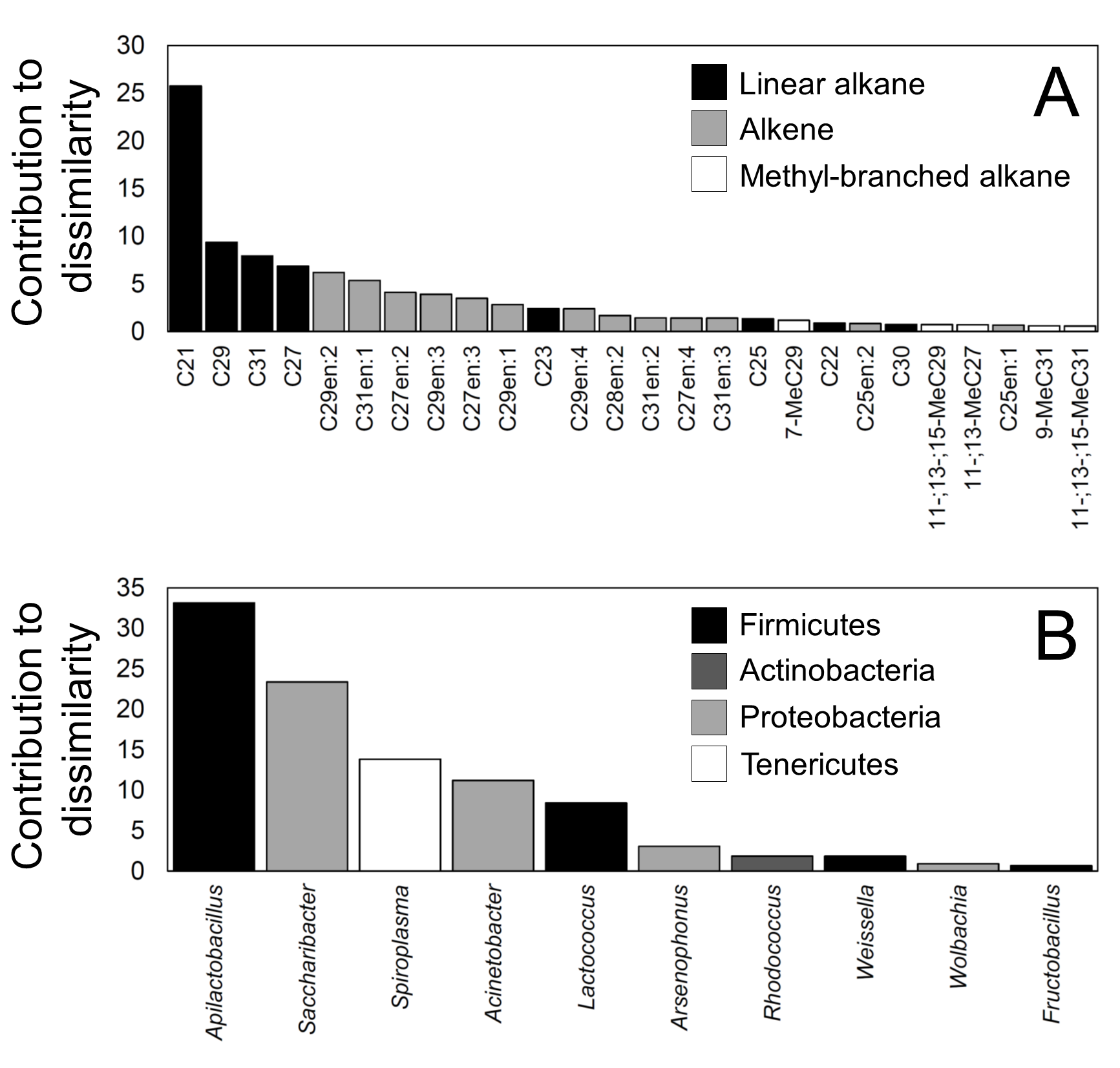

Supplement: Supplementary file 3 — Figure S2. SIMPER contributions to nest‐nest dissimilarity of (A) the 10 most important CHC compounds and (B) the 10 most important bacterial ASVs. [file ECE3-15-e71519-s003.tif]
