# Supplementary material for: The Significance of Genetic Relatedness and Nest Sharing on the Worker‐Worker Similarity of Gut Bacterial Microbiome and Cuticular Hydrocarbon Profile in a Sweat Bee
Source: Ecol Evol. 2025 Jun 9;15(6):e71519. doi: 10.1002/ece3.71519 (PMC12146657; doi:10.1002/ece3.71519)
Supplement: Supplementary file 4 — Table S1. The eight microsatellite markers and primers used in this study. [file ECE3-15-e71519-s001.docx]

**Supporting Information**

**Table S1.** The eight microsatellite markers and primers used in this study.

| **Locus** | **Reference** | **Dye** |
| --- | --- | --- |
| **Multiplex 1** |  |  |
| LHMS10 | Kukuk et al., 2002 | FAM |
| rub73 | Soro & Paxton, 2009 | FAM |
| rub02 | Soro & Paxton, 2009 | HEX |
| rub72 | Soro & Paxton, 2009 | ROX |
| **Multiplex 2** |  |  |
| rub37b | Soro & Paxton, 2009 | HEX |
| rub30 | Soro & Paxton, 2009 | FAM |
| rub77 | Soro & Paxton, 2009 | CY3 |
| LM27 | Paxton et al., 2003 | HEX |

**References**

Kukuk, P. F., Forbes, S. H., Zahorchack, R., Riddle, A., & Pilgrim, K. (2002). Highly polymorphic microsatellite markers developed for the social halictine bee *Lasioglossum (Chilalictus) hemichalceum*. Molecular Ecology Notes, *2*(4), 529-530.

Paxton, R. J., Arévalo, E., & Field, J. (2003). Microsatellite loci for the eusocial *Lasioglossum malachurum* and other sweat bees (Hymenoptera, Halictidae). Molecular Ecology Notes, 3(1), 82-84.

Soro, A., & Paxton, R. J. (2009). Characterization of 14 polymorphic microsatellite loci for the facultatively eusocial sweat bee *Halictus rubicundus* (Hymenoptera, Halictidae) and their variability in related species. Molecular Ecology Resources, *9*(1), 150-152.
