# Supplementary material for: The Significance of Genetic Relatedness and Nest Sharing on the Worker‐Worker Similarity of Gut Bacterial Microbiome and Cuticular Hydrocarbon Profile in a Sweat Bee
Source: Ecol Evol. 2025 Jun 9;15(6):e71519. doi: 10.1002/ece3.71519 (PMC12146657; doi:10.1002/ece3.71519)
Supplement: Supplementary file 5 — Table S2. The five genera of bacteria likely representing the core microbiome of H. scabiosae . [file ECE3-15-e71519-s002.docx]

**Supporting Information**

**Table S2.** The five genera of bacteria likely representing the core microbiome of *H. scabiosae.*

| **Genus** | **Number of bees in which it occurred** | **Abundance (%) range** | **Mean abundance (%) where occurring** |
| --- | --- | --- | --- |
| *Acinetobacter* | 55/90 | 1-53% | 13.5% |
| *Saccharibacter* | 75/90 | 1-93% | 24% |
| *Apilactobacillus* | 89/90 | 1-98% | 50.6% |
| *Lactococcus* | 39/90 | 1-68% | 12.5% |
| *Spiroplasma* | 30/90 | 1-75% | 26.2% |
| *Rhodococcus* | 23/90 | 1-18% | 4.3% |
